# The Economic Benefits Resulting from the First 8 Years of the Global Programme to Eliminate Lymphatic Filariasis (2000–2007)

**DOI:** 10.1371/journal.pntd.0000708

**Published:** 2010-06-01

**Authors:** Brian K. Chu, Pamela J. Hooper, Mark H. Bradley, Deborah A. McFarland, Eric A. Ottesen

**Affiliations:** 1 Lymphatic Filariasis Support Center, The Task Force for Global Health, Decatur, Georgia, United States of America; 2 Global Community Partnerships, GlaxoSmithKline, London, United Kingdom; 3 Rollins School of Public Health, Emory University, Atlanta, Georgia, United States of America; University of Oklahoma Health Sciences Center, United States of America

## Abstract

**Background:**

Between 2000–2007, the Global Programme to Eliminate Lymphatic Filariasis (GPELF) delivered more than 1.9 billion treatments to nearly 600 million individuals via annual mass drug administration (MDA) of anti-filarial drugs (albendazole, ivermectin, diethylcarbamazine) to all at-risk for 4–6 years. Quantifying the resulting economic benefits of this significant achievement is important not only to justify the resources invested in the GPELF but also to more fully understand the Programme's overall impact on some of the poorest endemic populations.

**Methodology:**

To calculate the economic benefits, the number of clinical manifestations averted was first quantified and the savings associated with this disease prevention then analyzed in the context of direct treatment costs, indirect costs of lost-labor, and costs to the health system to care for affected individuals. Multiple data sources were reviewed, including published literature and databases from the World Health Organization, International Monetary Fund, and International Labour Organization

**Principal Findings:**

An estimated US$21.8 billion of direct economic benefits will be gained over the lifetime of 31.4 million individuals treated during the first 8 years of the GPELF. Of this total, over US$2.3 billion is realized by the protection of nearly 3 million newborns and other individuals from acquiring lymphatic filariasis as a result of their being born into areas freed of LF transmission. Similarly, more than 28 million individuals already infected with LF benefit from GPELF's halting the progression of their disease, which results in an associated lifetime economic benefit of approximately US$19.5 billion. In addition to these economic benefits to at-risk individuals, decreased patient services associated with reduced LF morbidity saves the health systems of endemic countries approximately US$2.2 billion.

**Conclusions/Significance:**

MDA for LF offers significant economic benefits. Moreover, with favorable program implementation costs (largely a result of the sustained commitments of donated drugs from the pharmaceutical industry) it is clear that the economic rate of return of the GPELF is extremely high and that this Programme continues to prove itself an excellent investment in global health.

## Author Summary

Lymphatic filariasis (LF), commonly known as ‘elephantiasis’, is one of the world's most debilitating infectious diseases. In 83 countries worldwide, more than 1.3 billion people are at risk of infection with an estimated 120 million individuals already infected. A recent publication reviewing the health impact of the first 8 years of the Global Programme to Eliminate Lymphatic Filariasis (GPELF) demonstrated the enormous health benefits achieved in populations receiving annual mass drug administration (MDA), as a result of infection prevented, disease progression halted, and ancillary treatment of co-infections. To date, however, no studies have estimated the economic value of these health benefits, either to the individuals or the societies afflicted with LF. Our study estimates that US$21.8 billion will be gained among individuals benefitting from just the first 8 years of the Global Programme, and an additional US$2.2 billion will be saved by the health systems of endemic countries. Treating endemic populations is possible at very low cost – particularly because of the generous drug donations from two pharmaceutical companies – but results in enormous economic benefits. Findings from this study yield a much clearer understanding the GPELF's full economic impact and strengthen the conviction that it is a ‘best buy’ in global health.

## Introduction

As a leading cause of permanent and long-term disability worldwide, the parasitic infection lymphatic filariasis (LF) imposes a severe physical and socioeconomic burden on 1.3 billion at-risk persons in 83 endemic countries. An estimated 120 million people are already infected with LF, with about 40 million suffering from overt clinical disease manifested as painful severe swelling due to lymphedema (generally an accumulation of lymphatic fluid in the limbs) and hydrocele (fluid accumulation in the scrotal sac). To rid the world of this debilitating disease, the Global Programme to Eliminate Lymphatic Filariasis (GPELF) was begun in 2000 to guide endemic countries in implementing single-dose, once-yearly mass drug administration (MDA) using a combination of either albendazole+ivermectin or albendazole+diethylcarbamazine (DEC) for an anticipated 4–6 years. Use of this effective strategy for LF elimination has become feasible because of the drug donations of albendazole and ivermectin from their pharmaceutical manufacturers, GlaxoSmithKline and Merck & Co., Inc, respectively.

Over the first 8 operational years of the GPELF (2000–2007), more than 1.9 *billion* MDA treatments were administered to approximately 570 million individuals in 48 countries. This significant programmatic achievement has resulted in a notably beneficial impact on the health of endemic populations [1]. More than 6 million cases of hydrocele and 4 million cases of lymphedema have been prevented, resulting in over 32 million disability-adjusted life years (DALYs) averted and numerous quality-of-life benefits attained.

What remains relatively undefined, however, is the *economic* significance of these achievements. Specifically, how much financial cost and loss of income is prevented over the lifetimes of individuals protected from LF due to the first 8 years of the GPELF? And, what cost savings do national health systems realize from the reduction in LF infection and morbidity?

To date, few attempts have been made to examine LF from an economic perspective, particularly on a global level. Such data, however, is invaluable to policymakers, public health administrators, and program funders who may already be convinced that LF is a ‘best buy’ in global health but who lack precise estimates to support their conviction. This study offers such an economic analysis and estimates that following the first 8 years of the GPELF, US$21.8 billion of economic benefits will be gained by LF infected and non-infected individuals in MDA-treated areas, in addition to US$2.2 billion in health systems savings. Furthermore, though this economic assessment has not included the value of the many ancillary benefits on other concurrent infections that are effectively treated by the anti-LF drug regimens, it still leads to a far better understanding of the GPELF's true overall impact on one of society's most debilitating and widespread tropical diseases.

## Methods

### Data Sources

Key data sources are listed below, though specific sources are cited throughout the paper:

1. *LF at-risk, infected, and treated population estimates* are taken from The World Health Organization's *Weekly Epidemiological Record* and WHO Annual Reports between 2000–2008 [2], [3], [4], [5], [6], [7], [8].
2. *Health impact estimates* are taken from *The Global Programme to Eliminate Lymphatic Filariasis: Health Impact after 8 Years* [1].
3. *Direct treatment costs* are based on published literature (cited as presented) in relation to medicine prices gathered from Health Action International and Management Sciences for Health [9], [10].
4. *Indirect loss of labor estimates* are based on published literature as cited.
5. *Wage and income estimates* come from the International Labour Organization's *LABORSTA* database [11], the World Bank's *World Development Indicators Online* [12], and minimum wage estimates from the International Labour Organization [13] and the US Department of State's *Country Reports on Human Rights Practices 2008* [14].
6. *Official currency exchange and inflation rates* are from the International Monetary Fund's *World Economic Outlook 2008* database [15].

### Population Groups Analyzed for Economic Benefits from the GPELF – the “*Benefit Cohort Population*”

For this analysis, two broad groups of individuals are recognized as economically benefitting from the MDA treatment given during the first 8 years of the GPELF:

- Those protected from acquiring infection (and subsequent disease [specifically, hydrocele and/or lymphedema]);
- Those already infected but protected from disease progression.

These two groups can be segmented into four sub-populations (detailed below and summarized in **Table 1**); together, they constitute the “*benefit cohort population*”.

**Table 1:** Sub-populations of the “Benefit Cohort Population.”

Population Group	Subgroup	Definition
**1. Individuals protected from acquiring infection (and subsequent disease)**	a. Newborns	Babies born into MDA treated areas and whose entire lives are protected from potential LF infection and morbidity
	b. Other individuals protected from infection	Individuals who would have acquired infection but are protected because of interrupted transmission of LF resulting from MDA
**2. Individuals infected with LF but protected from progression of disease**	a. Subclinical disease	Patients with subclinical infection at the time of MDA who are protected from progression to clinical disease as a result of MDA
	b. Clinical disease	Individuals with clinical disease at the time of MDA who are either protected from worsening of their disease or actually undergo improvement as a result of MDA

Population estimates for each sub-population were calculated using the same base figures and key assumptions as described previously [1]; namely that 10% of the at-risk population is actually infected with LF, that this ratio would remain constant in the absence of MDA, and that the relative frequency of the clinical disease syndromes will also remain stable among those infected individuals.

#### Individuals protected from acquiring infection (and subsequent LF disease)

- **Newborns in MDA treated areas who are protected from infection over their lifetimes:** The number of babies born into LF treatment areas who likely would have become infected in the absence of MDA was calculated for *each country* covered by the GPELF between 2000–2007 based on the rates of surviving newborns, the levels of infection in at-risk populations, and the decreases in post-MDA infection exposure rates [1], [16]. These calculations resulted in the number of protected newborns being 6.6 million.
  Of all these babies who were protected from LF infection, an estimated 12.5% would have progressed to lymphedema and 20.8% to hydrocele; the remaining 66.7% would have had subclinical disease [1]. *For this study, it is assumed that only individuals with clinical disease (lymphedema or hydrocele) would have incurred any economic burden*. As previously published, an estimated 1.4 million cases of hydrocele and 0.8 million cases of lymphedema would have been averted in newborns between 2000–2007 in MDA treated areas [1].
- **Other individuals protected by MDA from acquiring LF infection:** In the absence of MDA, approximately 10% of the at-risk population is infected with LF [1]. To maintain this steady-state proportion in a *dynamic* population, non-infected individuals must continue to acquire infection through LF transmission at a rate sufficient to ‘replace’ those who leave the population (i.e. through death) each year. The size of this benefit population group is therefore equal to the number of infected patient deaths in the year; this total was calculated by multiplying the number of infected individuals who either had clinical disease or were expected to progress to clinical disease by the age- and country-specific mortality rates derived from the World Health Organization's *Life Tables for WHO Member States* [17].Between 2000–2007 in the populations covered by the GPELF, over 550,000 infected individuals who either had clinical disease or were expected to progress to clinical disease died; therefore, the same number of *replacement* infections would have been expected to occur over the same time-period to maintain the overall steady-state infection ratio in the at-risk population. However, because of MDA, these *replacement* individuals will be *protected* from acquiring infection and thus will accrue the benefits of averting clinical disease. As described elsewhere [1], the full protective benefits of MDA are likely not attained immediately after the first MDA, so calculations are based both on the numbers of people treated in each country each year and also on the assumption (derived from available transmission studies [1]) that only 50% would be protected after the first round of MDA treatment, 75% after the second, 87.5% after the third, 94% after the fourth, and 100% after the fifth. As a result, an estimated 480,000 individuals were protected from acquiring infection (and subsequent clinical disease) between 2000–2007.

All the protected individuals in these two ‘benefiting populations’ need not themselves have been directly treated during the MDA, as high MDA coverage in at-risk populations will drastically reduce the rate of transmission and, therefore, infection in untreated individuals as well [18], [19]. Indeed, reports from the World Health Organization do indicate high MDA coverage averaging more than 70% overall, with several regions and countries covering more than 90% [2], [3], [4], [5], [6], [7], [8].

#### Individuals already infected with LF but protected by MDA from progression of disease

- **Individuals with subclinical disease at the time of MDA:** Previous studies have shown that approximately two-thirds of individuals infected with LF will have subclinical disease [16] and about half of these are expected to progress to overt clinical disease in their lifetimes [1]. In order to remain conservative for the present analysis, it is estimated that MDA halts disease progression in only 50% of *those who would have progressed to clinical disease* [20]— and that disease is apportioned as described previously: 62.5% being hydrocele, 37.5% being lymphedema.

Since this study assumes that the only individuals incurring economic costs due to LF are those with clinical disease, the only individuals with subclinical disease whose benefits from MDA are tallied in this analysis are those who would have been expected to progress to clinical disease. Previous estimates are that 9.4 million subclinical cases were prevented from progressing to hydrocele and lymphedema between 2000–2007 [1].

- **Individuals with clinical disease at the time of MDA.** Of the 10% of the at-risk population who are infected, approximately one-third has overt clinical disease— again, with the majority of those manifesting hydrocele (62.5%) and the remaining, lymphedema. It was previously estimated that between 2000–2007, approximately 570 million at-risk individuals, including 57 million with LF infection, received MDA [1]; therefore, roughly 19 million individuals with overt clinical disease received MDA.It is still uncertain to what extent MDA improves morbidity in those already suffering from hydrocele or lymphedema, but recent studies provide preliminary evidence of the positive effects of repeated rounds of MDA on the progression or even reversibility of LF morbidity. Specifically, MDA has been shown to alleviate the number of acute ADL episodes associated with LF by 59–88% after just two rounds of annual MDA with DEC with and without albendazole [21], [22], [23]. The effects of MDA on chronic disease, however, are more uncertain. A study in Papua New Guinea resulted in complete reversal of 87% of hydrocele and 69% of leg lymphedema cases after 5 annual rounds of DEC+ivermectin or DEC alone [24], and studies in Indonesia [25], [26] and Tanzania [27] using DEC, provide evidence of an improvement or complete disappearance of clinical manifestations by 62–90% after 2–4 rounds of annual MDA. However, other studies have failed to show such significant clinical benefits from MDA [28], [29], [30]. While evidence of acute and chronic disease regression using a combination of ivermectin+albendazole or ivermectin alone is even less well documented, a recent report from Tanzania indicates that MDA (using albendazole+ivermectin) lessens the frequency and severity of ADL episodes by a significant degree. The same report also finds that approximately 15% of hydrocele cases and 98% of lymphedema cases had shown improvement after 4 annual rounds of MDA treatment using ivermectin+albendazole [31].Because of these uncertainties, the base analysis of this study used a low-end estimate of 50% reversal in the frequency of acute ADL episodes. For chronic disease, a reasonably conservative estimate was used – 10% of hydrocele cases and 15% of lymphedema cases were considered reversible after 5 rounds of MDA involving either DEC or ivermectin in the treatment regimen. However, in order to take into account the uncertainties of the outcomes of MDA on pre-existing clinical morbidity, a sensitivity analysis was also conducted ranging from 0% reversal to 90% reversal as per the lower and upper boundaries cited by the literature.

### Calculating the Total Economic Benefits of the GPELF

The previous section defined a 4-part *benefit cohort population* as the group of protected individuals who will realize economic benefits as a result of MDA activity between 2000–2007. The *total* economic impact of the GPELF, however, extends over a much longer period than these first 8 years because protection from LF infection or disease progression is a lifelong benefit. It is therefore necessary to aggregate the total economic benefit gained over the *projected remaining lifetime* of the *benefit cohort population*.

**Figure 1:**
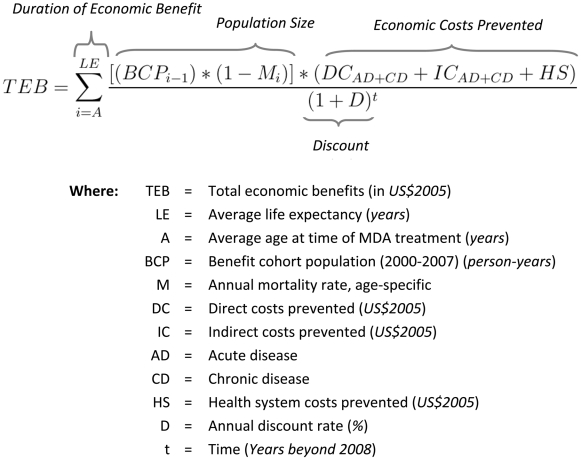
General formula for calculating economic benefits. The formula was applied and calculated independently for each country to accommodate country-specific differences in several key parameters (i.e. life expectancy, mortality rate, direct and indirect costs). All calculated costs and benefits are discounted by 3% per year to the base year of 2008.

To estimate this total, a general formula **(** **Figure 1** **)** was applied and calculated independently for each country to accommodate country specific differences in several key variables (life expectancy, mortality rate, direct and indirect costs). All calculated costs and benefits are discounted to the base year of 2008.

#### Duration of economic benefits

**Figure 2:**
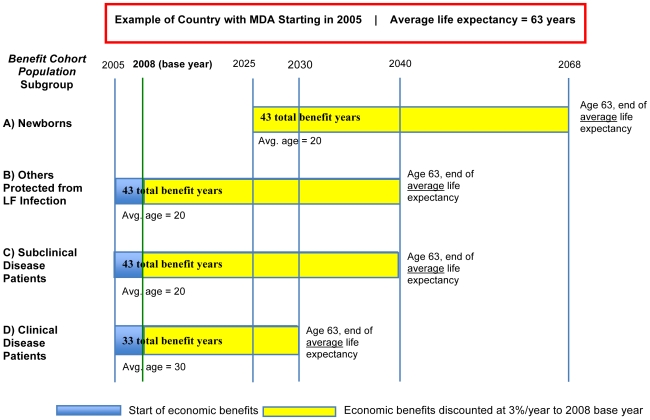
Duration of economic benefits. Economic benefits are calculated only for the *benefit cohort population* receiving MDA between 2000–2007; however, the benefits are gained until the end of their lifetime. For modeling purposes, single average ages were used to encompass the entire age range of individuals in each population subgroup, realizing that some individuals will be above this average age at the time of treatment, and some below. The size of each subgroup decreases each year based on country and age-specific mortality rates.

The duration of economic benefits depends on the age of onset of clinical disease (assumed as 20 years old [1] in each country), the average life expectancy (differing by country), and the age at which an individual received MDA treatment. In this model, for each subgroup population, a *same single average age* for each country was used to encompass the entire age range of individuals within the *benefit cohort population* at the time of treatment, with the recognition that some of those receiving treatment will be younger than the average age and some older (**Figure 2**).

- **Newborns** (Group 1a, **Table 1**) did not actually receive MDA treatment but are considered protected from the time of birth once transmission has been interrupted; thus their average age at the time of treatment is 0. However, because the average age of clinical disease onset is 20 years old [1], *benefits for newborns do not accrue until 20 years after birth* because on average, no clinical disease and hence, economic costs will be incurred before that age;
- For **other individuals protected from LF infection** (Group 1b), MDA treatment is estimated to occur at 20 years of age on average.
- **Infected individuals with** ***subclinical disease*** (Group 2a) are also estimated to be 20 years old on average when they receive MDA. Though subclinical infection is common in early childhood, this model assumes that the average age of treated (and thus protected) subclinical patients is 20 years.
- **Infected individuals with overt** ***clinical disease*** (hydrocele or lymphedema, Group 2b) are estimated to be 30 years old on average when they receive MDA. This estimate implies that clinical-disease patients have been living with their condition for an average of 10 years, since onset of clinical disease is taken as 20 years of age.

#### Population size

This study projects the total economic impact of the first 8 years of the GPELF by aggregating the economic benefits over the lifetime of the 2000–2007 *benefit cohort population*. To remain consistent with this study's goal of only estimating the GPELF's economic achievements following these 8 years, no projections are made for the growth of MDA treatments after 2007 or any resulting economic benefits to new individuals treated beyond the *benefit cohort population*.

Year-to-year, the size of the *benefit cohort population* will decrease because of country-specific mortality and average life expectancy. Because the model assumes this non-static population over time, the economic-benefit denominator must be analyzed in *person-years*, which is the sum of each year lived by each individual in the *benefit cohort population*. **Table 2** shows that the first 8 years of the GPELF will provide over 1.1 billion person-years of economic benefit during the lifetime of the 31.4 million individuals in the *benefit cohort population*.

**Table 2:** Benefit Cohort Population: Individuals and person-years.

Population Group	Population Subgroup	Benefit Cohort Population Size (2000–2007) (*millions*)	Average Age of MDA Treatment^1^	Average Years of Economic Benefit^2^	Person-Years (Lifetime) (*millions*)^3^
**1. Protected from acquiring infection (and subsequent disease)**	*a) Newborns*	*2.2*	*-*	*43*	*83.8*
	*b) Protected from infection*	*0.5*	*20*	*43*	*15.7*
	***Subtotal***	**2.7**	***-***	***-***	**99.5**
**2. Protected from disease progression**	*a) Subclinical morbidity*	*9.4*	*20*	*43*	*388.6*
	*b) Clinical morbidity*	*19.3*	*30*	*33*	*626.6*
	***Subtotal***	***28.7***	***-***	***-***	***1,015.3***
**Total**		**31.4**	**-**	**-**	**1,114.8**

1 Newborns, although not actually treated with MDA, are assumed protected from infection at the time of birth and protected from clinical disease from 20 years of age.

2 Based on average life expectancy of 63 years, weighted by country-specific rates and *Benefit Cohort Population* in each country.

3 Sum of each year lived by each individual in the Benefit Cohort Population. Equal to (*Benefit Cohort Population*)×(Average Years of Economic Benefit), adjusted for annual mortality.

#### Economic costs prevented

Economic costs are comprised of *direct treatment costs* and *indirect labor costs*. Economic costs are further segmented in each sub-population group (**Table 1**) by disease type (acute or chronic) and morbidity type (hydrocele or lymphedema). Key model assumptions and estimates, weighted by country–specific rates, are summarized in **Table 3**.

- ***Direct treatment costs*** refer to costs (e.g., for medicines, consultation fees, transport, food, accommodation) that are incurred when an individual with clinical morbidity seeks treatment.

**Table 3:** Epidemiological and cost estimates used in the Economic Benefit Model.

Parameter Type	Acute or Chronic Disease	Associated Cost-Type	Rate or Proportion	Regional Variation	Hydrocele Avg. Estimate^1^		Lymphedema Avg. Estimate^1^		Sources, Key Assumptions
*Epidemiological*	*Acute Disease*	*Direct and Indirect*	% of clinical LF patients with ADL	Global estimate^2^	70%	[45–90%]	95%	[90–95%]	[33], [35], [39], [40], [41], [42], [43], [44], [45]
			% of patients with ADL seeking treatment	Global estimate, India excepted^3^	65%	[55–70%]	65%	[55–70%]	[33], [34], [35], [42]
			# of ADL episodes per patient (w/o MDA)	Global estimate^2^	2	[0–7]	4	[0–7]	[33], [35], [39], [40], [41], [42], [43], [44], [45]
			*% of ADL episodes prevented by MDA*	Global estimate^2^	*50%*	*[15–88%]*	*50%*	*[15–88%]*	[21], [22], [23], varies by MDA round
		*Indirect Cost*	Avg. duration of ADL episode (days)	Global estimate^2^	4	[0–9]	4	[0–9]	[33], [35], [39], [40], [41], [42], [43], [44], [45]
			% of work hours lost per day due to ADL	Global estimate^2^	75%	[50–93%]	75%	[50–93%]	[40], [42], [44], [51]
	*Chronic Disease*	*Direct and Indirect*	% of Chronic disease patients seeking treatment	Global estimate, India excepted^3^	40%	[20–50%]	50%	[30–55%]	[34], [36], [37], [38]
			*% of Chronic disease patients benefiting from MDA*	Global estimate^2^	*10%*	*[0–87%]*	*15%*	*[0–69%]*	[24], [25], [26], [27], [28], [31], [55], varies by MDA round
		*Indirect Cost*	% of work hours lost per day due to chronic disease	Global estimate^2^	15%	[13–17%]	20%	[15–22%]	[36], [37], [40]
*Cost*	*Acute Disease*	*Direct Cost*	Avg. treatment cost per episode	Country-specific estimate^4^	$1.5	[$0.25–$5.20]	$1.5	[$0.25–$5.20]	[9], [10], [33], [34], [35], [36], [37]
	*Chronic Disease*	*Direct Cost*	Avg. treatment cost per year	Country-specific estimate^4^	$2.9	[$0.55–$10.05]	$4.3	[$0.85–$15.00]	[9], [10], [33], [34], [35], [36], [37]
	*Acute and Chronic Disease*	*Indirect Cost*	Avg. wage per day	Country-specific estimate^4^	$1.05	[$0.30–$5.60]	$1.05	[$0.30–$5.60]	[11], [12], [13], [14]
	*Chronic Disease*	*Indirect Cost*	Work days per year	Global estimate^2^	300	[300–365]	300	[300–365]	Assuming 6 workdays/week

1 Weighted average over all GPELF countries.

2 Global estimate indicates a standard rate or proportion was utilized for each GPELF country. This is primarily due to a lack of supporting country-specific data.

3 Indicates a standard rate or proportion was utilized for each GPELF country with the exception of India where more primary data was available and suggested estimates differ from other GPELF regions.

4 Estimates are country-specific and gathered from public online international database sources.

Patients seeking treatment for acute inflammatory attacks caused by LF usually receive pain relieving and anti-inflammatory medicines, with or without systemic antibiotics [32]. Chronic disease sufferers may also seek care following bouts of severe pain and swelling and receive a similar treatment package. Chronic patients may also purchase bars of soap in accordance with prevailing lymphedema management strategies [32].

Median international reference prices for a course of amoxicillin, ibuprofen, and paracetamol were collected from Health Action International [9] and Management Science for Health [10] to approximate public and private sector costs of medicines across GPELF countries. Primary data show that for individuals seeking treatment at health facilities for LF, medicines, on average, comprise 50% of the total treatment cost, consultation fees 30%, and transport, food, and accommodation the remaining 20% [33], [34], [35], [36], [37]. For self-treatment individuals, only the medicine costs were attributed to total treatment costs.

***Acute disease*** refers to periodic, recurring attacks of acute adenolymphangitis (ADL), defined by signs and symptoms of pain, tenderness, local swelling, and warmth in the groin or limbs with constitutional symptoms such as fever, nausea, and vomiting [35], [38], [39].

Approximately 70% of hydrocele patients experience at least 1 ADL episode per year with an average of 2. For lymphedema patients, almost 95% experience at least 1 episode with an average of 4. The number of episodes for both morbidities, however, can be up to 7 or higher. Each ADL episode lasts on average 4 days although the duration can range to 9 or more days [33], [35], [39], [40], [41], [42], [43], [44], [45].

The proportion of individuals with ADL episodes who seek treatment – whether at a health facility, traditional healer, or through self-treating with medicine – ranges from 55% to 70% depending on the country and region. Similarly, the preferred treatment source and related costs are highly region-specific. In WHO-AFRO, self-treatment and traditional healers may be used in 70% of cases, leading to a weighted average cost across all sources of US$0.90 per ADL episode treated. In comparison, WHO-SEARO has an average weighted cost of US$1.40 per episode treated, largely due to the higher cost and proportion of treatment seeking in urban areas and private health facilities in India [33], [34], [35], [42]. WHO-WPRO has an even greater average weighted cost of $4.90 due to higher wages and standard of living costs in this region. Across all GPELF countries, the overall weighted average of treatment seeking behavior and costs for ADL was 65% and $1.50 respectively.

***Chronic disease*** refers to individuals with overt clinical disease in individuals with hydrocele and/or lymphedema. All population groups with economic benefits from MDA in this study are assumed to have (or would otherwise have acquired) chronic disease but only a proportion of these chronic disease patients incur acute ADL episodes.

The percentage of chronic disease patients who seek treatment is also highly dependent on the country/region, severity of disease, and availability of treatment. On average, this study conservatively assumes that 30% of hydrocele and 35% of lymphedema patients seek treatment, although in India these proportions are estimated at 60% and 65%, respectively, based on the available literature. These estimates are also weighted over time with the assumption that almost all chronic disease patients will seek treatment in the early years of disease morbidity but will reduce their frequency over the long-term. Chronic disease patients are assumed to seek treatment on average twice a year with lymphedema patients seeking and spending slightly more. On average, treatment seeking hydrocele patients will spend US$2.90 and lymphedema patients US$4.30 per year for their chronic conditions [34], [36], [37], [38].

*Hydrocelectomy* (surgery to repair hydrocele) costs are included in the chronic disease direct cost calculation. The proportion of total direct costs related to hydrocelectomies, however, is very small because of the relatively low frequency of hydrocelectomies; hydrocele patients often have poor access to surgery facilities and are further deterred by the restrictive costs of the procedure.

- ***Indirect labor costs*** refer to income lost as a result of reduced work hours and economic activity due to LF morbidity. For women, economic activity also includes time spent on domestic chores because an opportunity cost of income-generating activity is implied. As in previous LF studies and burden of disease analyses, indirect cost estimates were calculated using the human capital approach, which presumes total cost and lost output are equal to the income foregone as a result of illness [46], [47].

Approximating the income for individuals with LF is difficult because the majority of this population is comprised of subsistence farmers who do not participate in the formal labor market. A variety of methods in valuing working time have been incorporated in economic analyses of populations with similar tropical diseases (malaria, trachoma, onchocerciasis), including the examination of minimum wages [48], average value added per agricultural worker [49], and proxies from prior studies in similar settings [50]. Based on these methods, the combination of 3 wage sources was used in this paper for best estimates of a fair market value of time for an agricultural worker with LF infection: (1) The International Labour Organization's *LABORSTA* database which lists average wages for agricultural field workers [11]; (2) The World Bank's World Development Indicators Online which lists the average value added per agricultural worker [12]; (3) The International Labour Organization's Minimum Wages Database and US Department of State's Country Reports on Human Rights Practices which list minimum wages by occupation including agricultural and low-skilled workers [13], [14].

For countries listed by one or more sources, the lowest wage amount was used to ensure a conservative estimate. For countries not listed by any of the three sources, the lowest amount within the same region was used as a proxy. In this paper, it is assumed that all individuals with or at-risk of LF would have been economically active otherwise and would work 300 days per year.

***Acute disease:*** Acute ADL episodes are severely debilitating,with studies in India showing total economic disability for the entire duration of the episode in 81–87% of cases versus 34–37% of controls [40], [51]. Based on these and additional case-control studies, the present analysis assumes 75% of time spent on economic activity is lost due to acute disease during an ADL episode [42], [44].

***Chronic disease:*** Although LF chronic disease is less debilitating than acute ADL episodes, chronic disease patients still work fewer hours than equivalent non-LF workers. While the amount of disability is strongly correlated to the degree of disease, hydrocele patients are estimated to work 15% less time and lymphedema patients 20% less on average [36], [37], [40].

- ***Health system costs:*** Comprehensive assessment of economic costs and benefits must also include the potential savings to the health system since decreased LF infections reduce medical treatments needed. To estimate these patient-service savings, country specific costs were gathered from the WHO-CHOICE database, recording costs for a 20-minute visit to a primary health center having a 50% regional coverage [52]. These costs were then multiplied by the number of individuals benefiting from MDA, the percentage that seek treatment in public health facilities, and the average number of visits per year.

***Cost Standardization:*** To standardize the comparison of prices and wages over different time periods, all estimates when necessary were adjusted to 2005 values (to correspond to external supporting World Bank data) using national consumer price index (CPI) data [15]. Estimates were then converted from local currencies to US dollars using official average 2005 exchange rates [15].

### Discount Calculation

For this study, 2008 was used as the base year for calculating economic benefits. When calculating future benefits, however, it is necessary to discount values to a net present value (NPV) under the economic principle that a dollar earned in the present is worth more than one earned in the future. Therefore, all annual accumulated cost savings beyond 2008 are discounted at 3% per year in accordance to guidelines set by WHO-CHOICE [53].

### Cost-Benefit Calculations

GPELF program costs can be compared with the economic benefits calculated in this study through a cost-benefit analysis to evaluate the efficiency and practicality of implementing the Global Programme. Estimating program costs, however, is not the intent of this study and such data was, therefore, sourced through our previously published work [54]. On a macro-level, no study has yet been conducted to estimate the total cost of the GPELF over its first 8 years and as a result, a broader programmatic cost-benefit analysis cannot be calculated. It is possible, however, to estimate the cost-benefit on an individual-level using per person costs calculated in a multi-country study of national MDA program costs for LF including training, mapping, mobilization, distribution, monitoring, and surveillance [54]. In this study, *Goldman et al.* analyzed both the average annual *economic* cost per person treated (i.e. including the implied costs of donated materials and drugs – set at US$0.19+$0.0019 for shipping per 400mg tablet of albendazole and US$1.50+$0.0018 per 3mg tablet of ivermectin) and also the *financial* cost per person treated (i.e. excluding the costs of the donated materials and drugs) from data collected through questionnaires and adjusted for LF-specific activities. Donated ivermectin is used in combination with donated albendazole in areas co-endemic for onchocerciasis in Africa plus Yemen. DEC, which is not donated, is used in combination with donated albendazole in all other countries and must be purchased by national programs.

In terms of per person economic benefits, the total economic benefits estimated over one year in this study was divided by the total number of people treated with MDA in that same year. For this analysis, per person economic benefits were only calculated for the 7 countries whose program costs were also evaluated in *Goldman et al.'s* study. Cost-benefit was then measured using benefit-cost ratios (BCR), which is the per person treated benefit divided by the per person treated cost. For standardization purposes, the BCR reflects costs, benefits, and currencies adjusted to the year of the most recent MDA round in *Goldman et al.'s* study.

## Results

### Economic Benefits to the *Benefit Cohort Population*

During the first 8 years of LF MDA, the Global Programme delivered nearly 2 billion treatments and reached almost 570 million individuals in 48 of the 83 identified endemic countries (**Table 4**). As a result of these program achievements, 31.4 million individuals – defined in this study as the *benefit cohort population* – will gain economic benefits over their lifetime from averting direct treatment costs and indirect lost-labor costs. Of these 31.4 million individuals in the *benefit cohort population*, 2.7 million (8.6%) would have acquired LF and subsequently progressed to clinical disease but were protected from infection altogether because of interruption of the transmission cycle by MDA. This group comprises the proportion of *newborns* (2.2 million) that are protected by virtue of being born in MDA-treated areas, as well as *other individuals in the general population* (0.5 million) protected because LF transmission has been interrupted.

**Table 4:** GPELF MDA treatments (2000–2007).

WHO Region	GPELF Countries (2000–2007)	Individuals Treated with MDA (*Millions*)	Treated Individuals Infected with LF (*Millions*)^1^	Benefit Cohort Population (*Millions*)
**AMRO**	Brazil, Dominican Republic, Guyana, Haiti	2.2	0.2	0.1
**AFRO**	Benin, Burkina Faso, Cameroon, Comoros, Ghana, Kenya, Madagascar, Mali, Niger, Nigeria, Senegal, Sierra Leone, Tanzania (incl. Zanzibar), Togo, Uganda	51.2	5.1	2.9
**EMRO**	Egypt, Yemen	2.7	0.3	0.2
**WPRO**	American Samoa, Cambodia, Cook Islands, Fed. States of Micronesia, Fiji, French Polynesia, Kiribati, Marshall Islands, Malaysia, Niue, Papua New Guinea, Philippines, Samoa, Tonga, Tuvalu, Vanuatu, Vietnam, Wallis and Futuna	17.4	1.7	1.0
**SEARO**	Bangladesh, India, Indonesia, Maldives, Myanmar, Nepal, Sri Lanka, Thailand, Timor-Leste	494.4	49.4	27.2
**All Regions**	**48 total countries**	**567.9**	**56.8**	**31.4**

1 Assumed that 10% of at-risk population is actually infected with LF [1].

The remaining 28.7 million (91.4%) individuals are those who were already infected at the time of MDA treatment but benefit from halted disease progression. This population group comprises individuals at the *subclinical* disease stage (9.4 million, [29.9%]) who avoid clinical disease altogether and individuals at the *clinical disease* stage (19.3 million [61.6%]) whose conditions may improve following MDA.

As seen in **Table 5**, the efforts in reaching and administering MDA to such a large population have produced extraordinary economic benefits over the first 8 years of the GPELF. An estimated US$21.8 billion will be saved over the lifetimes of the 31.4 million individuals who have or would have acquired clinical disease during this timeframe. This total amount results from summing the direct treatment costs ($1.4b) and indirect lost wages ($20.4b) prevented over the lifetime of each of the population groups under the assumptions and estimates previously outlined in **Table 3**. Direct costs for acute disease were calculated based on the aggregate number of ADL episodes expected in the absence of MDA and the average cost incurred per episode. Chronic disease direct costs were derived from the percentage and total number of patients seeking treatment multiplied by the average cost spent per treatment. Indirect costs for both acute and chronic disease were calculated by accruing the equivalent workdays lost to LF and multiplying this total by the average daily wage. All average costs and rate of disease estimates were weighted annually by country-specific estimates and with respect to total number of person-years.

**Table 5:** Total costs prevented over lifetime of Benefit Cohort Population.

			*Direct Costs Prevented*		*Indirect Costs Prevented*		
Population Group	Population Subgroup	Benefit Cohort Population (*millions*)	Acute Disease (US$MM)	Chronic Disease (US$MM)	Acute Disease (US$MM)	Chronic Disease (US$MM)	Total Costs Prevented (US$MM)
**1. Protected from acquiring infection (and subsequent disease)**	*a) Newborns*	*2.2*	*$71*	*$6*	*$207*	*$1,444*	*$1,727*
	*b) Protected from infection*	*0.5*	*$24*	*$2*	*$75*	*$532*	*$633*
	***Subtotal***	***2.7***	***$95***	***$8***	***$282***	***$1,975***	***$2,360***
**2. Protected from disease progression**	*a) Subclinical morbidity*	*9.4*	*$584*	*$49*	*$1,765*	*$12,146*	*$14,544*
	*b) Clinical morbidity*	*19.3*	*$528*	*$89*	*$1,596*	*$2,698*	*$4,911*
	***Subtotal***	***28.7***	***$1,112***	***$138***	***$3,361***	***$14,844***	***$19,455***
**Total**		**31.4**	**$1,207**	**$146**	**$3,643**	**$16,819**	**$21,815**

On average, each individual of the *benefit cohort population* will avoid nearly $700 in LF-associated costs that would have accrued over his/her lifetime. This equates to the amount earned for 19 working days per person-year, thus preventing the loss of approximately 6.3% of annual income (**Table 6**). These sums and averages are even greater when considered in a single year-to-year perspective, since beyond 2008, each year of economic benefit is discounted by 3% per year.

**Table 6:** Total costs prevented per individual of the Benefit Cohort Population.

Population Group	Population Subgroup	Benefit Cohort Population (*millions*)	Total Costs Prevented (US$MM)	Lifetime Benefit per Individual	Avg. Annual Lost Work Days Prevented	Avg. % of Annual Lost Work Days Prevented
**1. Protected from acquiring infection (and subsequent disease)**	*a) Newborns*	*2.2*	*$1,727*	*$783*	*20*	*6.7%*
	*b) Protected from infection*	*0.5*	*$633*	*$1,319*	*39*	*13.1%*
	***Subtotal***	**2.7**	**$2,360**	**$879**	**23**	**7.7%**
**2. Protected from disease progression**	*a) Subclinical morbidity*	*9.4*	*$14,544*	*$1,552*	*36*	*12.1%*
	*b) Clinical morbidity*	*19.3*	*$4,911*	*$255*	*8*	*2.5%*
	***Subtotal***	***28.7***	***$19,455***	***$679***	***19***	***6.2%***
**Total**		**31.4**	**$21,815**	**$696** 1	**19** 1	**6.3%** 1

1 Weighted average of all *Benefit Cohort Population* subgroups.

**Table 6** also shows that the infected patient sub-population groups (i.e. *clinical* and *subclinical*) have the greatest total lifetime benefits based on their larger proportion of the total *benefit cohort population*. On a per person lifetime average, however, *subclinical* (Group 2a) and ‘*other protected individuals*’ (Group 1b) benefits are larger.

**Figure 3:**
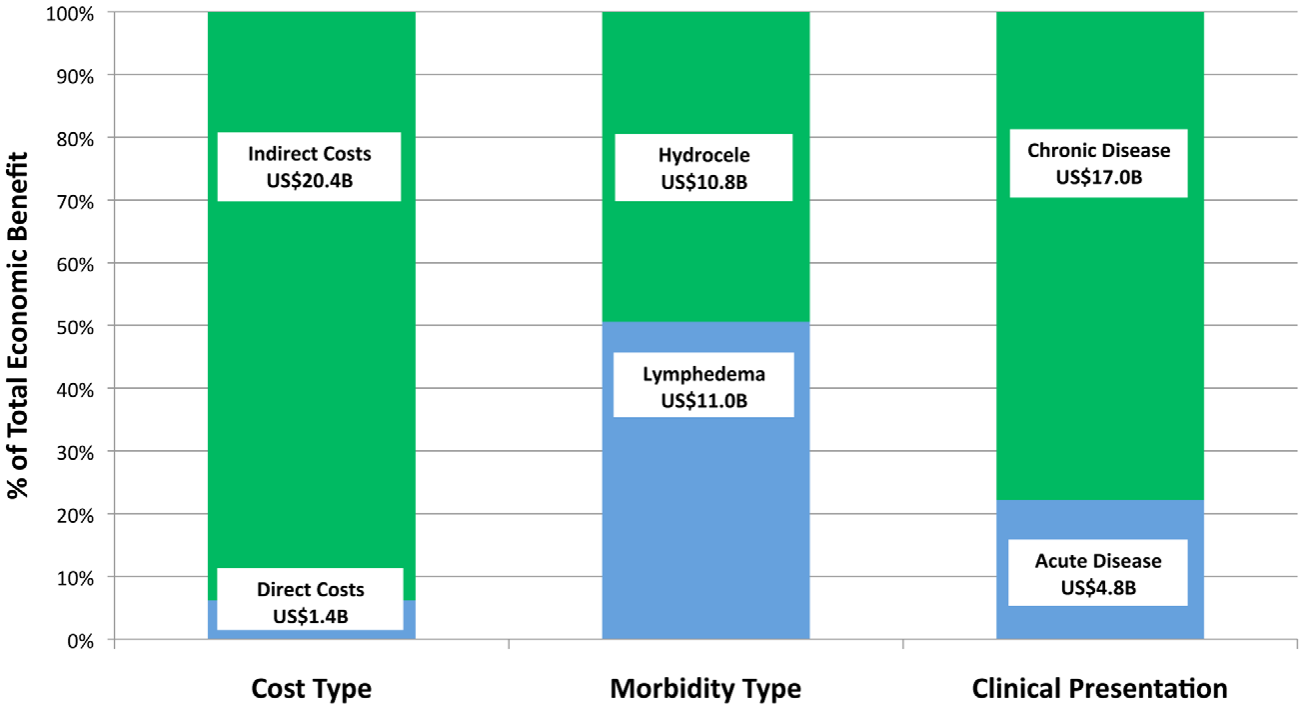
Total Economic benefits by category. The total economic benefit for individuals (i.e. excluding health system savings) of US$21.8 billion can be further analyzed by cost type, morbidity type, and clinical presentation.

**Figure 3** highlights the total economic benefit segmented by cost, morbidity, and clinical presentation.

#### Identifying benefits by cost type

Approximately 94% of the total economic benefits were due to the prevention of indirect costs of lost working time and, therefore, output and income (**Figure 3** **, Section A**). The lower proportion of direct treatment costs (6%) was attributable to the low frequency of treatment seeking behavior and inexpensive medicine packages relative to the day-to-day accumulation of lost income from reduced economic activity.

#### Identifying benefits by morbidity type

Economic benefits accruing to populations protected from hydrocele are approximately equal to those from lymphedema (**Figure 3** **, Section B**). The estimated higher proportion of clinical disease patients with hydrocele (62.5%) compared to lymphedema (37.5%) offsets the greater average disability of lymphedema patients in terms of their ADL frequency, ADL duration, and percentage of work time lost due to disease.

#### Identifying benefits by clinical presentation

Preventing chronic disease accounts for about 78% of the total economic benefits – not unexpected given the long-term disabling nature of LF (**Figure 3** **, Section C**). Acute episodes generally affect individuals for only 8–12 days a year, whereas the chronic condition is a perpetual disability. Moreover, studies investigating the effects of DEC on individuals with overt clinical disease show greater evidence towards the lessening of ADL episodes than the reduction or reversal of the chronic condition. If future studies can demonstrate unequivocal benefits of MDA for chronic disease patients, its proportion of economic benefits will be even higher.

#### Identifying benefits per region

**Table 7** highlights the regional variation in cost savings among GPELF programs. Much of the difference in per person benefits can be attributed to higher average costs and wages outside of the AFRO and SEARO regions. The total GPELF benefits, however, are heavily concentrated in SEARO and in particular in India, which comprised over 75% of all individuals treated during the 8-year period.

**Table 7:** Lifetime economic benefits by region.

WHO Region	Total Lifetime Benefit (US*$*MM)^1^	Lifetime Benefit per Patient	Avg. Annual Lost Work Days Prevented	Avg. % of Annual Lost Work Days Prevented
**AMRO**	$183	$1,446	20	6.7%
**AFRO**	$1,288	$439	23	7.5%
**EMRO**	$146	$922	20	6.6%
**WPRO**	$2,128	$2,186	18	6.0%
**SEARO**	$18,070	$665	19	6.2%
**All** 2	**$21,815**	**$695**	**19**	**6.3%**

1 Does not include health system benefit.

2 Weighted average over all WHO regions.

### Economic Benefits to Health Systems

**Figure 4:**
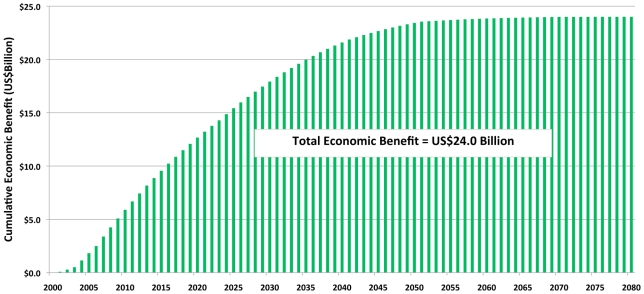
Cumulative economic benefits resulting from the first 8 years of the GPELF. Total economic savings to individuals and health systems accumulate throughout the *benefit cohort population's* lifetime.

Economic benefits to national health systems resulting from reduced LF infections derive particularly from patient service costs averted in the public sector. Approximately US$2.2 billion in health system costs will be saved over the lifetime of the *benefit cohort population* (**Table 8**). Combined with the US$21.8 billion savings for individuals, the total economic benefit following the first 8 years of the GPELF is estimated at an extraordinary US$24.0 billion (**Figure 4**).

**Table 8:** Health system economic benefits.

WHO Region	Benefit Cohort Population (*millions*)	% Seeking Treatment at Gov't Primary Health Center^1^	Cost per 20 Minute Outpatient Visit to Government Primary Health Center^2^	Total 2008 Health System Cost Averted (US$MM)	Total Lifetime Health System Costs Averted (US$MM)
**AMRO**	0.1	52%	$3.7	$0.2	$4.3
**AFRO**	2.9	52%	$2.4	$2.7	$53.5
**EMRO**	0.2	52%	$2.1	$0.1	$3.8
**WPRO**	1.0	52%	$4.0	$1.4	$39.8
**SEARO**	27.2	52%	$2.2	$81.0	$2,085.7
**All Regions**	**31.4**	**52%**	**$2.3**	**$85.5**	**$2,187.1**

1 Weighted between acute and chronic disease patients across all GPELF countries within WHO region.

2 Using WHO-CHOICE estimates. Weighted average for all GPELF countries within region.

### Sensitivity Analyses

This overall economic analysis of the GPELF's first 8 years does have notable limitations due to the lack of regionalized primary data concerning both epidemiological and socioeconomic factors associated with LF. Indeed, two sections of the model remain with particular uncertainty: 1) *The degree to which MDA can reverse or return an individual with overt clinical disease to regular productivity*, and 2) *The relationship between hours lost and output/productivity lost due to disease*. The total estimated economic impact is particularly sensitive to these variables because of the large number of clinical disease patients receiving MDA and the high proportion of total prevented costs that come from indirect labor costs. As a result, sensitivity analyses were carried out to assess the range of economic benefits that can be achieved under varying estimates of each variable. Secondary sensitivity analyses investigating the frequency and duration of ADL episodes, and direct treatment costs indicated less variability of economic benefits when adjusting these parameters.

#### Chronic disease regression and reversal with MDA

As noted previously, there is considerable debate about the degree of regression of existing hydrocele and lymphedema following MDA treatment. Estimates range from no regression at all to complete reversal of 87% of hydrocele cases and 69% of leg lymphedema cases after 5 annual rounds of DEC with ivermectin or DEC alone [24]. Other estimates from related studies also lie within this range [25], [26], [27], [28], [31], [55], which is therefore used as the lower and upper boundaries for the sensitivity analysis. A linear relationship will also be assumed – i.e. that a 50% reversal of chronic disease would lead to an average 50% increase in the potential lost aggregate working hours for a chronic disease patient over his or her entire remaining lifetime.

**Table 9** compares the degree of chronic disease reversal to the total economic benefit gained by the clinical disease population. In our model, US$21.8 billion would be saved based on 10% of hydrocele and 15% of lymphedema cases considered *curable* through MDA. If these estimates even double to 20% and 30%, respectively, the total amount rises to US$24.4 billion, equivalent to approximately 7.2% of annual income for inidividuals of the *benefit cohort population*. Under the maximum assumptions based on the data of *Bockarie et al.* [24], total economic benefits would be a staggering US$37 billion or 11.0% of individual annual income. With such a varying degree of potential economic impact that is also likely dependent on parasite and MDA regimen type, a better understanding of the true relationship between antifilarial drug treatment and filarial morbidity is needed. In particular, prospective studies with rigorous case definitions, close clinical monitoring, control groups, and outcomes focused on clinical morbidity rather than microfilaria prevalence alone [32], would strengthen our understanding of this relationship. Nevertheless, that there exists scientific data supporting chronic disease regression and reversal remains uncontroversial and further studies in this regard will be essential to determining the full economic impact of MDA on the clinical manifestations of hydrocele and lymphedema.

**Table 9:** Sensitivity analysis for chronic disease reversal following MDA.

Selected Studies	Hydrocele Reversal/ Improvement	Hydrocele Patients (n)	Lymphedema Reversal/ Improvement	Lymphedema Patients (n)	Estimated Economic Benefit Based on Study Parameters (US$MM)	Avg, % of Annual Lost Income (Work Days) Prevented
Ciferri 1960, Dunyo 2000, Das 2003^1^	0%	37	0%	26–48	$18,890	5.5%
Partono 1985	-	-	71%	49	$27,590	8.0%
Partono 1981	-	-	75%	20	$28,010	8.1%
Mackenzie 2009	15%	13	98%	62	$31,020	9.0%
Meyrowitsch 1996	67%	60	39%	26	$31,700	9.2%
Bockarie 2002	87%	105	69%	90	$37,390	11.0%
**Base model estimates**	**10%**	**-**	**15%**	**-**	**$21,815**	**6.3%**

1 No change or results considered insignificant.

#### Indirect productivity loss due to clinical LF

Our analysis calculates indirect costs based on the equivalent hours and resulting wages lost from economic activity. This approach, however, ignores how much *output* and *productivity* are actually lost as a result of fewer work hours. In a study of Indian weavers, the reported productivity gap between LF and non-LF individuals was 27% [56], which is markedly higher than the 15–20% time difference used for measuring indirect costs from chronic disease in this study. In defense of the analysis, time valuation was utilized on its own because supporting primary data on productivity burden was absent from the literature. Moreover, *Ramu et al.'s* study [56] evaluated the productivity of weavers whereas compared to farmers, output is not predicated on seasonal and environmental factors that would cause increased correlation variation between time and output. Nevertheless, this distinction between *time* and *productivity* burden would likely only underestimate the true disability and loss of earning power for individuals with clinical disease.

At our base rate of 15–20% productivity loss averted, the total lifetime benefit for the *benefit cohort population* is US$21.4 billion. By increasing this rate to 30%, the total lifetime economic benefit rises almost 75% to US$36.4 billion, equivalent to 10.6% of individual annual income. This high sensitivity to chronic indirect costs indicates that additional research on the actual productivity and economic output burden of LF, rather than time alone, will bring significant value to developing a more precise economic benefit estimate in the future.

Similarly, chronic disease indirect costs are particularly sensitive to the average earnings of individuals with LF. This study chose to estimate conservatively by basing a wage on the minimum income amounts listed for agricultural workers using 3 separate database sources – the ILO's *LABORSTA* database [11], the World Bank's *World Development Indicators Online* [*12*], and minimum wage estimates from the International Labour Organization [13] and the US Department of State's *Country Reports on Human Rights Practices 2008* [*14*]. If instead only World Bank wages were used, the average wage, weighted across all regions, would increase from $1.05 to $1.40 and the total economic benefit estimated in this model would rise from $21.4 billion to $28.8 billion, which is 8.4% of individual annual income. If only ILO wages were implemented in the model, the average wage would be $1.50 and the total economic benefit $30.7 billion or 9.0% of individual annual income. Additionally, LF patients may be employed in occupations earning more than subsistence farmers (e.g. weaving, mining, fishing) and therefore suffer a higher opportunity cost of illness. Average incomes are also generally higher in urban areas, where up to a third of the LF burden in India exists [46], [57]. More socioeconomic research of LF patients will be necessary to yield greater accuracy of the opportunity cost of the disease and indirect economic benefits of the GPELF.

#### Acute episodes

Acute ADL episodes are notably debilitating and clearly inhibit economic activity. Therefore, the number and duration of annual ADL episodes prevented has an impact on both direct and indirect economic benefits. In this study's base model, it is estimated that individuals with hydrocele incur on average 2 ADL episodes per year and for lymphedema patients, 4 episodes per year. Previous studies have shown the annual incidence can vary from 1 to 8 [33], [45], [58], resulting in a range of total economic benefits of $19.0 billion to $25.6 billion (5.5% to 7.4% of individual annual income). The economic impact is also dependent on the average duration of ADL episodes, which can last from 2 to 16 days. The upper end of this range would lead to economic benefits totaling $32.2 billion (9.4%) with a more modest increase in average duration of 6 and 8 days resulting in benefits of $23.3 billion and $25.0 billion, respectively (6.8%–7.3%). On the lower end, assuming ADL episodes last an average of only 2 days decreases the total economic benefit to $19.8 billion (5.8%).

#### Direct treatment costs

The results from Figure 3 show that prevention of direct treatment costs constituted only 6.2% of the total economic benefit. Therefore, while previous research indicates a large variance in the cost, source, and frequency of treatment, the economic benefit outcome is not as sensitive to these variables as with chronic indirect costs. While there are reported cases of LF patients in private hospitals or using multiple treatment sources spending up to $40 per ADL episode [31], [51] or $200 per year [37] for chronic disease treatment, these instances represent extreme outliers given that the mean costs associated with the reviewed literature typically range from $1–$5. If we were to double the average prevented costs of treatment for ADL episodes, hydrocele, and lymphedema, the total economic benefit would marginally rise to $22.9 billion (6.7% of individual annual income). Tripling the average treatment cost would result in an economic benefit of $26.2 billion (7.6%), which is a far less elastic outcome than when varying the parameters of chronic indirect costs or even the frequency of ADL episodes.

### Cost-Benefit Analysis

*Goldman et al.* found the average annual *economic* cost per person treated (i.e. including the implied costs of donated materials and drugs – set at US$0.19+$0.0019 for shipping per 400mg tablet of albendazole and US$1.50+$0.0018 per 3mg tablet of ivermectin) ranged from US$0.40 in Philippines to $5.82 in Tanzania. The average annual *financial* cost per person treated (i.e. excluding the costs of the donated materials and drugs) ranged from US$0.06 in Burkina Faso to US$1.34 in Haiti [54].

One-year economic benefits per person treated in this study ranged from US$1.00 in Burkina Faso to US$4.56 in the Dominican Republic. **Table 10** compares these economic benefit estimates with the economic and financial costs from *Goldman et al.'s* study to calculate country-specific BCRs. The *economic* cost BCR for the three African countries using the ivermectin+albendazole regimen are lower (0.21–0.37) than those in countries using DEC+albendazole (1.23–8.59). Since the drugs, however, are available at no cost to the GPELF, BCRs calculated using *financial* costs are more favorable, ranging from 1.64 in Egypt to 18.07 in the Philippines.

**Table 10:** Country-specific benefit-cost ratios.

				Economic Benefit - Financial Cost^1^		Economic Benefit - Economic Cost^2^	
Country	Year	MDA Round	Avg. Economic Benefit per Person Treated (*1-year*)^3^	Financial Cost per Person Treated^4^	Benefit-Cost Ratio (*1-year*)	Economic Cost per Person Treated^4^	Benefit-Cost Ratio (*1-year*)
**Burkina Faso** *	2002	2	$1.00	$0.06	16.67	$4.82	0.21
**Ghana** *	2002	2	$1.82	$0.17	10.72	$4.88	0.37
**Tanzania** *	2003	4	$0.99	$0.26	3.81	$4.53	0.22
**Dominican Republic**	2003	2	$4.56	$0.87	5.24	$1.56	2.92
**Egypt**	2001	2	$1.64	$1.00	1.64	$1.34	1.23
**Haiti (Leogane)**	2002	3	$2.84	$1.30	2.18	n/a	-
**Haiti (Milot)**	2002	1	$3.60	$1.10	3.27	n/a	-
**Philippines**	2003	3	$3.43	$0.19	18.07	$0.40	8.59

1 Financial cost does not include the cost of ivermectin and albendazole, which are both donated. DEC must be purchased by national programs and is therefore included as a financial cost. Ivermectin is used in combination with albendazole in areas co-endemic for onchocerciasis in Africa plus Yemen. DEC, which is not donated, is used in combination with albendazole in all other countries and must be purchased by national programs.

2 Economic cost includes the implied cost of donated materials and drugs (Source: Goldman et al. (2007) [54]): US$0.19+$0.0019 for shipping per 400mg tablet of albendazole and US$1.50+$0.0018 per 3mg tablet of ivermectin.

3 Includes both individual and health system benefits. Currency is adjusted to match year of MDA round.

4 Source: Goldman et al. (2007) [54].

*Countries receiving the albendazole+ivermectin drug regimen.

## Discussion

LF is a pervasive, disabling disease whose importance is magnified by the fact that 1.3 billion people are at risk of infection in some of the poorest countries in the world. LF causes not only a severe physical burden on sufferers but also a considerable economic burden from both direct medical expenses and loss of income-generating activity. While precise data on the *economic burden* of LF morbidity have been scarce [59], it was the earlier estimate of disease impact resulting in US$1 billion in lost productivity each year in India alone [46] and another $1 billion combined for the endemic countries in Africa [60] that contributed to the World Health Assembly's resolution for the elimination of LF and WHO's subsequent creation of the *Global Programme to Eliminate Lymphatic Filariasis* (GPELF) [61].

The present study constitutes the first attempt to quantify the principal *economic benefit* of the first 8 years of the GPELF and, as such, complements an earlier analysis on the health impact of these first 8 years [1]. It conservatively estimates that in 2008 alone, over US$775 million of direct and indirect patient costs were averted as a result of MDA in 48 endemic countries. Of the 570 million *individuals* treated in the MDAs, 31.4 million either had clinical disease or would have acquired clinical disease during these 8 years. In the *entire lifetimes* of these 31.4 million people, costs totaling of US$21.8 billion (an average of nearly US$700 per person) will be averted. On a per person-year basis, this translates to approximately 6.3% of one's average *annual* income after future discounting.

In the absence of MDA, much of the economic burden can be attributed to indirect costs in the form of lost labor time. ADL episodes exacerbate the chronic pathology of lymphedema and hydrocele, and can lead to total disability for the entire duration of 81–87% of acute attacks [40], [51]. The economic burden is also greater should ADL episodes occur more frequently during the critical planting seasons for agricultural workers, which evidence from some literature suggests is the case [41], [44], [62], [63].

Despite this greater severity and incapacitation of ADL, it is the lifelong disabling nature of the chronic conditions that makes LF such an economically crippling disease. In calculating the total indirect costs of chronic disease, this study estimated that over 1 billion working hours each year would have been foregone without MDA. At an estimated 15–20% reduction in daily work hours, approximately 6–8% of equivalent workdays are lost annually to chronic disease. Related studies from Ghana and India indicate 3.8% and 7.0% of all potential male labor inputs, respectively, were lost annually as a result of chronic LF [38], [40].

Direct treatment costs, while much less than the indirect cost of lost labor, are still significant. Direct costs are especially burdensome because an acute or chronic disease patient may need to borrow money to pay for treatment or, more commonly, must avoid treatment altogether because it is unaffordable. Estimates from the literature suggest that only 60–70% of ADL and 40–50% of chronic disease sufferers on average, sought treatment (including self-treatment) – highlighting a tradeoff between financial and physical burden. In this study, the average treatment expense for an ADL episode is estimated at $1.40, which is almost 1.5 times greater than the estimated average daily income. Treatment in private facilities or using multiple treatment sources, however, can range up to 10 or more times this amount. In reality, costs can vary even more as some cases in India and Tanzania reported spending nearly $40 per ADL episode [31], [51]. The same extent of the range of direct costs can be found for chronic disease patients. A study in eastern India reported treatment costs upwards of $200 per year [37]. While treatment seeking behavior is likely to be higher in a patient's early years with chronic disease, it is still reasonable to assume that direct treatment costs will be sustained in a patient's later years because the chronic manifestations themselves will progress with age. Along with this progression, the need for other forms of treatment and pain management will remain.

LF is recognized as one of the most important neglected tropical diseases (NTDs), which are often characterized as diseases of poverty. Indeed, it is clear that the considerable losses in labor inputs from LF over a prolonged period of time make it all the more difficult for endemic areas to escape from such a poverty trap without MDA intervention. Although chronic disease patients may develop coping strategies to adapt to their condition and regain some time spent in economic activity, many do so at the expense of lower-earning jobs that require less physical activity [38], [64]. In Tanzania, it is roughly reported that patients who were primarily fishermen lose about 53% of their income each month due to chronic LF disease [65]. Those with severe morbidity may be confined to the home and forced to give up income-generating activity altogether.

Proponents of the friction-cost method of indirect cost calculation would argue that substitute labor could replace the lost inputs and outputs of an LF patient [66]. However, with most LF patients working outside the formal economy, other family members would have to act as substitute labor, which often subtracts the same proportion of household income. For example, a study on the economic burden of malaria found household members more likely to care for the patient than act as substitute labor, particularly when skilled labor is involved; when substitution did happen, output was not perfectly replaced [67]. Friction-cost theorists also argue that lost hours can be made up by extra productivity during non-sick hours; however, reduced labor inputs in time-sensitive activities such as agricultural planting cannot be so easily replaced later on. LF studies indicate that it may even be necessary for sick individuals to hire temporary workers to replace their labor, thus exacting an even greater indirect financial burden on the patient.

In analyzing the economic impact for the 31.4 million *benefit cohort population*, this study estimates that individuals receiving MDA before infection or at the subclinical disease stage have a much higher average lifetime and annual economic benefit than individuals already manifesting clinical disease (**Table 6**). With advanced stages of hydrocele and lymphedema posing even greater risks of physical and economic disability [39], MDA at the pre-infection or pre-clinical disease stage is critical, particularly in high-transmission areas. Furthermore, high coverage rates in areas undergoing MDA allow a subgroup of untreated individuals to be protected from infection, subsequent clinical disease, and the incurrence of economic costs as a result of reduced rates of LF transmission. The extension of benefits to individuals beyond those directly receiving MDA or infected with LF accentuates the wider community economic impact of the GPELF.

This broader impact also includes financial savings to national health systems as a result of decreased need for patient services associated with LF morbidity. Using WHO-CHOICE's valuation of health center outpatient visits [52], MDA from 2000–2007 will lead to an estimated economic benefit of approximately US$2.2 billion over the same timeframe as calculated for the 31.4 million *benefit cohort population*. Such significant savings are particularly critical for resource constrained health systems and primary health centers operating beyond capacity. The economic benefits to health systems are arguably even greater than the estimate presented in this analysis. This model did not account for the effect of MDA on decreasing the need for hydrocele surgeries and lymphedema morbidity-support services because accurate regionalized data on the extent of averting these specific provider costs is limited. From the scarce literature available, hospitals in Tanzania, coastal Kenya, and northern Ghana have reported that 15–25% of all surgeries performed were for hydrocele [68], [69] and that establishing a single lymphedema treatment clinic in Haiti can cost the health system US$8,000 [70]. In India, the additional cost of implementing filariasis control programs at the primary health center (PHC) level was estimated at approximately US$800 per PHC per year [71]. Limiting the future need for such services will bring sizable cost savings for both national filariasis control programs and health systems, which further underscores the GPELF's societal economic impact.

### Cost-Benefit Calculations

In **Table 10**, the BCRs calculated with *economic* costs are low, particularly for the African countries using the ivermectin+albendazole regimen. In reality, however, the whole foundation of the GPELF is the long-term, sustained commitment of drug donations offered by GlaxoSmithKline for albendazole and Merck & Co., Inc. for ivermectin for as long as needed until LF is eliminated [72], [73]. Because of this commitment, governments and donors will never have to finance these costs themselves; indeed, without these commitments there would be no GPELF. Therefore, understanding the *financial* costs (i.e. excluding the costs of the donated materials and drugs) to the GPELF is more relevant for policy- and decision-making than is the analysis of economic costs. When comparing *financial costs* to *economic benefits*, then, **Table 10** shows very favorable BCRs, including up to 18.07 in the Philippines.

Whether examining financial or economic costs, the BCR becomes larger in the years beyond the recommended 5 rounds of MDA to achieve lifetime protection from LF. The *Goldman et al.* study [54] showed strong evidence that costs decrease after the initial year of implementation and after 5 rounds of MDA, the drug costs and majority of program activities would arguably subside dramatically as well. In the Philippines for example, by conservatively extrapolating the initial year's annual *economic* cost over 5 years, the cost to *lifetime* benefit ratio indicates that a $1 investment leads to a sizable return of $60 per individual treated. While this analysis does not account for costs following the 5^th^ round of MDA (e.g. post-MDA surveillance), it can be reasonably assumed that the BCR would still remain very significant. Indeed, even if annual *economic* costs were to persist at an equivalent rate for an additional 10 to 15 years, the economic rate of return per person treated is still approximately $20–$30 for every $1 invested.

Comparing the cost-benefit of the GPELF to that of other NTD programs is difficult because there are few directly comparable analyses, particularly at a global level. A review of the African Programme for Onchocerciasis Control (APOC) projected an economic rate of return of 6% to 17% but did not factor in the implied economic cost of the donated drug [74]. This finding is less than the drug-excluded LF cost-benefit estimate presented here, however, several economic benefits apart from onchocercal blindness prevention were not analyzed in the APOC review. Cost-benefit analyses for trachoma have focused almost exclusively on trichiasis surgery in a localized context [75]. A broader array of cost-benefit studies has been carried out for malaria, although with differing scopes and outcome goals, making it challenging to compare results across the same disease, let alone between malaria and LF. In a review of several malaria costing studies, the BCR ranged from 1.9 to 17.1 using a variety of human capital and burden of disease methodologies [76]. Other approaches assessing a more macroeconomic impact of malaria [77], [78] have yet to be applied to NTDs but future research into such cost-benefit applications will be critical for validating the investment of the GPELF and stimulating likeminded investigations for related global NTD programs.

There has been considerable movement – particularly in Sub-Saharan Africa – toward integrating preventive chemotherapy programs to target multiple NTDs (e.g. LF, onchocerciasis, schistosomiasis, soil-transmitted helminths [STH], trachoma) together. Although there is no clear verdict yet on the benefits of integrated NTD treatments versus standalone vertical programs, early assessments indicate potential savings of 25–47% for the entire group of NTDs can be achieved in Sub-Saharan Africa by packaging MDA interventions together [79]. These findings underlie an important concept of economic analysis, specifically that although an intervention (e.g. vertically integrated MDA programs for LF) may have a favorable BCR, there may be more *cost-effective* alternatives to achieving a similar *outcome* (e.g. the number of treatments administered, the total economic benefits of the GPELF). In this respect, the GPELF is well positioned to take advantage of synergistic opportunities with other disease program activities including vector control (malaria and dengue fever), surveillance (guinea worm, onchocerciasis), and distribution (integrated NTDs, Vitamin A) to maximize cost-effectiveness and economic impact [80]. Joint efforts with the private sector and drug development projects addressing improved sanitation and housing facilities could also contribute to greater cost-effectiveness for the GPELF [81]. While this study is not positioned to analyze cost-effectiveness in details, it is abundantly clear that under any joint or standalone scenario, the GPELF indeed represents an excellent buy in global health.

### Additional Economic Benefits of the GPELF

The prevention of LF infection and clinical disease has led to additional benefits that are difficult or impossible to quantify in monetary terms. The true economic value of the GPELF is, therefore, arguably much higher based on the numerous quality-of-life benefits achieved through clinical disease aversion, as well as the economic impact that MDA has on other diseases and syndromes related to LF.

#### Quality-of-life benefits

Quality-of-life benefits may relate only peripherally to the economic burden of the disease but may be equally as important as the costs included in the model due to their direct impact on patient livelihood. Social stigma is a very important consequence of LF morbidity. The ostracism and isolation that LF patients experience in their communities can lead to delayed treatment seeking; this results in faster progression to later stages of morbidity where the economic burden is even higher [82]. In organized labor, employers may fire patients with obvious morbidity due to decreased productivity or misunderstanding of disease etiology. Female patients are often not considered suitable for marriage if they have lymphedema, which heavily impacts social and economic status [59], [83], [84]. Similarly, males with hydrocele report difficulties in finding spouses, ridicule from community members, and various degrees of sexual dysfunction [85]. Schoolchildren are expected to stay home to care for a family member with LF who is experiencing an acute attack, and infected schoolchildren frequently miss school or drop out due to ostracism [86]. When LF impacts income-generating activity of the heads of households, children may be forced out of school and into labor at an early age. This absenteeism from school and eventual dropping out maintains the poverty cycle for affected families and has important implications for endemic communities as a whole [82]. The efforts of the GPELF in eliminating these devastating consequences of clinical disease have created enormous quality of life benefits that have undoubtedly led to a tremendous economic impact through enhanced productivity and community welfare.

The GPELF must also be recognized as more than just a MDA-based distribution program As such, the GPELF's ‘second pillar’ is to provide care and initiate strategies for the control of clinical morbidity [32]. In particular, compliance to GPELF activities based on personal hygiene management of lymphedema has caused tremendous improvements in the physical and mental well-being of chronic disease patients. These improvements surely produce an unquantifiable economic benefit and reinforce the notion that even if antifilarial drugs do not have a direct effect on clinical morbidity reversal, the GPELF has created other mechanisms for long-term increased productivity for overt clinical disease patients.

#### Economic impact on other LF syndromes and co-endemic diseases

MDA reduces the acquisition of other debilitating overt clinical LF manifestations such as chyluria and tropical pulmonary eosinophilia (TPE). While these other syndromes are less prevalent than hydrocele and lymphedema, their physical and resulting economic burden can be even more severe. Socioeconomic data concerning such LF-associated syndromes, however, is essentially absent and therefore currently unquantifiable. Also not considered quantitatively was the full economic impact of subclinical LF infection. By protecting individuals from *even reaching the subclinical level*, the GPELF will have garnered economic benefits from preventing the renal disease, lymphatic dilatation, and lymphatic dysfunction in subclinically infected patients [87], [88].

The GPELF's drug regimens also result in decreased economic costs for other diseases *besides* LF – including river blindness and scabies in Africa and intestinal parasites globally. Considering the disease burden of these three infections and their geographic overlap with LF, it is certain that an important reduction in these diseases is found in MDA treatment areas, resulting in health and economic benefits from prevention and diminution of stunting, anaemia, renal disease, and other complications [89], [90].

### Study Limitations

A narrow range of country-specific primary data somewhat limits the breadth of economic analysis presented in this paper; however, much of the prevailing literature originates from India and Sub-Saharan African countries where over 75% of the *benefit cohort population* resides. Of significance, there is scarce regional data regarding treatment-seeking behavior for LF patients, but because this is a direct cost input, more exact data would result in only marginal changes to the overall economic impact. Similarly, LF disease-specific parameters (e.g. ADL frequency and duration, lost workdays) were attributed a global standardized estimate due to a lack of regional data. Sensitivity analyses conducted earlier in this study presented the resulting economic impact under differing degrees of pathology and indeed, a clearer understanding of regional variability would enrich future economic analyses. Other variables such as wages, health system costs, and direct treatment costs, however, were able to be made region- and country-specific with the aid of international databases from the ILO, WHO, and World Bank.

### Economic Projections for the Future Impact of GPELF

Despite important limitations to our analysis, this study identifies a wide array of economic benefits that have resulted from the first 8 years of the GPELF – approximately US$21.8 billion of direct and indirect patient costs will be prevented in the lifetimes of more than 31 million individuals, US$2.2 billion of LF-associated patient services saved by national health systems over the lifetimes of the MDA-treated individuals, and additional quality-of-life benefits and treatment of co-morbidities such as STH that make the total economic value of the GPELF unquestionably far greater than the calculable estimate presented here.

**Figure 5:**
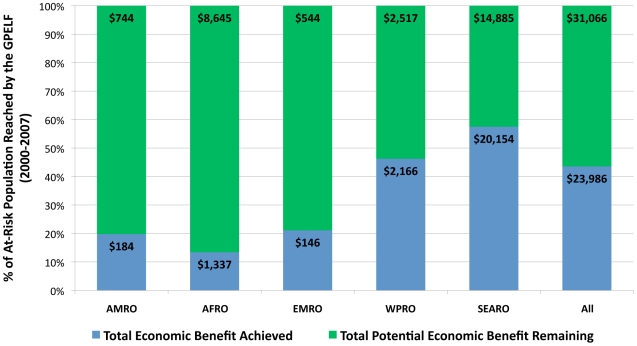
Potential economic impact of the GPELF. Indicates the economic benefit already achieved and the potential benefit remaining should the GPELF reach all endemic countries and at-risk populations.

These achievements notwithstanding, it is clear that the economic impact will be even greater when the GPELF reaches the remaining endemic countries and at-risk populations. Currently, the GPELF has extended to 48 of the 83 endemic countries and treated nearly 570 million individuals – approximately 44% of the ∼1.3 billion worldwide at-risk population [8]. Extrapolating this proportion with the US$24 billion lifetime economic benefit already achieved, the full *potential* economic benefit of the GPELF could be in excess of US$55 billion distributed over each of the endemic WHO regions (**Figure 5**).

Reaching the remaining at-risk individuals presents notable challenges especially since much of the population not yet reached resides in some of the poorest countries in Africa. Additional resources and economic empowerment will be necessary to assist these countries in implementing programs for LF elimination [91]. The expansion of the GPELF will therefore be a critical building block in this effort and also an important driver for increased attention to NTDs and the continuation of integrated NTD programs. The recognition of the sizable monetary benefit already achieved after 8 years provides new confidence that it is an investment well worth undertaking.

## Supporting Information

Alternative Language Abstract S1 Translation of the abstract into French by PJH.
(0.03 MB DOC)
