# Supplementary material for: The Economic Benefits Resulting from the First 8 Years of the Global Programme to Eliminate Lymphatic Filariasis (2000–2007)
Source: PLoS Negl Trop Dis. 2010 Jun 1;4(6):e708. doi: 10.1371/journal.pntd.0000708 (PMC2879371; doi:10.1371/journal.pntd.0000708)
Supplement: Alternative Language Abstract S1 — Translation of the abstract into French by PJH. (0.03 MB DOC) [file pntd.0000708.s001.doc]

**The Economic Benefits Resulting From The First 8 Years Of The Global Programme To Eliminate Lymphatic Filariasis (2000-2007)**

***Contexte :*** Entre 2000 et 2007 le Programme Mondial pour l’Élimination de la Filariose Lymphatique a délivré plus de 1,9 milliards de traitements à environs 600 millions d’individus à travers la distribution en masse annuelle de médicaments anti-filariose (albendazole, ivermectine, et diethylcarbamazine) à toutes les personnes à risque pendant 4 à 6 années. Quantifier les avantages économiques résultant de cette œuvre est important pour non seulement justifier les ressources investies dans le Programme Mondial mais aussi pour mieux comprendre l’impact global du Programme sur les populations endémiques les plus pauvres.

***Méthodologie :*** Pour évaluer les avantages économiques, le nombre de manifestations cliniques évité a été d’abord quantifié et les économies associées à cette prévention de la maladie ont été analysés en prenant en compte les coûts directs de traitement, les coûts indirects de temps de travail perdu, et les coûts liés au système de santé pour soigner les individus affectés. Plusieurs sources de données ont été examinées, y compris les publications et bases de données de l’Organisation Mondiale de la Santé, du Fonds Monétaire International, et de l’Organisation Internationale du Travail.

***Résultats :*** Les avantages économiques directs qui seront acquis au cours de la durée de vie de 31,4 millions d’ individus traités pendant les premières 8 années du Programme Mondial ont été estimés à US$21,8 milliards. Plus de US$2,3 milliards de ce total résultent de la protection de presque 3 millions de nouveau-nés et autres individus contre la filariose lymphatique par le fait d’être né dans les zones libérées de transmission de FL. De même, plus de 28 millions d’individus déjà infectés par la FL bénéficient de l’arrêt de progression de leur maladie grâce au Programme Mondial, résultant en un avantage économique pendant leur durée de vie d’approximativement US$19,5 milliards. En plus de ces avantages économiques pour les personnes à risques, les systèmes de santé dans les pays endémiques économisent approximativement US$2,2 milliards à cause de la diminution des services pour les patients associée avec la réduction de morbidité FL.

***Conclusions :*** Les distributions de masse des médicaments pour la FL offrent des avantages économiques importants. En outre, avec les coûts abordables de mise en œuvre du programme (dû aux engagements soutenus des dons de médicaments de l’industrie pharmaceutique), il est clair que le taux de retour économique du Programme Mondial est extrêmement élevé et que ce Programme continue prouver qu’il est un excellent investissement en matière de santé sur le plan mondial.
